# MYC Regulates a DNA Repair Gene Expression Program in Small Cell Carcinoma of the Ovary, Hypercalcemic Type

**DOI:** 10.3390/cancers17132255

**Published:** 2025-07-07

**Authors:** James R. Evans, Jing Wang, Cinthia N. Reed, Joy H. Creighton, Kaylee B. Garrison, Abigail N. Robertson, Ashley Lira-Rivera, Diondre’ D. Baisden, William P. Tansey, Rafet Al-Tobasei, Jessica D. Lang, Qi Liu, April M. Weissmiller

**Affiliations:** 1 Department of Biology, Middle Tennessee State University, Murfreesboro, TN 37132, USA; james.r.evans@vanderbilt.edu (J.R.E.); cinthia.n.reed@vanderbilt.edu (C.N.R.); jhc4y@mtmail.mtsu.edu (J.H.C.); kaylee.chisam1255@gmail.com (K.B.G.); arobert7@uab.edu (A.N.R.); al6v@mtmail.mtsu.edu (A.L.-R.); ddb6s@mtmail.mtsu.edu (D.D.B.); 2 Department of Cell and Developmental Biology, Vanderbilt University School of Medicine, Nashville, TN 37240, USA; william.p.tansey@vanderbilt.edu; 3 Center for Quantitative Sciences, Vanderbilt University Medical Center, Nashville, TN 37232, USA; jing.wang.1@vumc.org (J.W.); qi.liu@vumc.org (Q.L.); 4 Department of Biostatistics, Vanderbilt University Medical Center, Nashville, TN 37203, USA; 5 Department of Biochemistry, Vanderbilt University School of Medicine, Nashville, TN 37240, USA; 6 Department of Computer Science, Middle Tennessee State University, Murfreesboro, TN 37132, USA; rafet.al-tobasei@mtsu.edu; 7 Department of Pathology & Laboratory Medicine, University of Wisconsin-Madison, Madison, WI 53706, USA; jessica.lang@wisc.edu; 8 University of Wisconsin Carbone Cancer Center, University of Wisconsin-Madison, Madison, WI 53706, USA; 9 Center for Human Genomics and Precision Medicine, University of Wisconsin-Madison, Madison, WI 53706, USA

**Keywords:** MYC, DNA repair, SCCOHT, ovarian, SWI/SNF, BRG1, rhabdoid tumor, ATR, SNF5, *SMARCA4*

## Abstract

Background/Objectives: SCCOHT is an aggressive and often fatal cancer that belongs to the ~20% of cancers defined by mutations to subunits of the SWI/SNF chromatin remodeling complex. In SCCOHT, mutations to the *SMARCA4* gene, which encodes the SWI/SNF ATPase BRG1, are sufficient to impair SWI/SNF function. This single genetic lesion leads to a cascade of events that promote tumorigenesis, some of which may involve the intersection of SWI/SNF with oncogenic pathways such as those regulated by the *MYC* oncogene. In SCCOHT tumors and other cancers marked by SWI/SNF subunit mutation, MYC target genes are recurrently activated, pointing to a relationship between SWI/SNF and MYC that has yet to be fully explored. Methods: In this study, we investigate the contribution of MYC to SCCOHT biology by performing a combination of chromatin binding and transcriptome assays in genetically engineered SCCOHT cell lines, with subsequent validation using patient tumor expression data. Results: We find that MYC binds to thousands of active promoters in the BIN-67 SCCOHT cell line and that the depletion of MYC results in a broad range of gene expression changes with a notable effect on the expression of genes related to DNA repair. We uncover an MYC-regulated DNA repair gene expression program in BIN-67 cells that is antagonized by BRG1 reintroduction. Finally, we identify a DNA repair gene signature that is upregulated in SCCOHT tumors and in tumors defined by loss of the SWI/SNF subunit SNF5. Conclusions: Collectively, these data implicate MYC as a robust regulator of DNA repair gene expression in SCCOHT and lay a foundation for future studies focused on interrogating the relationship between BRG1 and MYC.

## Simple Summary

Small cell carcinoma of the ovary, hypercalcemic type (SCCOHT) is a rare and aggressive cancer with limited treatment options. Understanding the underlying biology of SCCOHT is critical to comprehending how this cancer is formed and identifying new ways to treat it. The aim of this study was to explore how MYC—a major oncogenic transcription factor with intimate ties to cancer—influences SCCOHT biology. Using engineered cell lines, we characterize the genes occupied by MYC and identify a cohort of genes activated by MYC that function in DNA repair and genome stability. We also discover a DNA repair gene signature that is overexpressed in SCCOHT tumor samples compared to normal ovary tissue, pointing to MYC as a key regulator of DNA repair gene expression in these cancers.

## 1. Introduction

SCCOHT is an aggressive and rare ovarian cancer [1] that usually occurs in early adulthood [2]. SCCOHT can be distinguished from other ovarian cancers based on multiple features, including age of onset, the presence of small closely packed round cells, and increased serum calcium levels [1,2,3]. The outcome for a patient with SCCOHT is often fatal, with a less than 20% two-year survival rate and recurrence of disease appearing in ~65% of cases [2,4]. The most effective treatment for SCCOHT includes chemotherapy and surgery followed by a single high-dose chemotherapy combined with stem-cell transplantation, but even this intense treatment regimen only moderately increases three-year survival rates [5].

SCCOHT cancers are part of the nearly 20% of all malignancies harboring mutations in SWI/SNF subunit genes (herein called “SWI/SNF-altered cancers”) [6]. SWI/SNF is a multi-subunit chromatin remodeling complex that regulates gene expression through controlling DNA accessibility. Mutations to SWI/SNF subunits occur in a broad range of cancers and are typically deleterious to subunit expression, suggesting that loss of SWI/SNF function promotes tumorigenesis [7,8]. The molecular pathogenesis of SCCOHT is overwhelmingly linked to loss-of-function mutations in the *SMARCA4* gene, which encodes the BRG1 subunit [9,10,11]. BRG1 is one of two mutually exclusive ATPases that are responsible for the ATP-dependent activity of SWI/SNF, but the second ATPase, *SMARCA2*, is not expressed in these cancers [12] making loss of BRG1 in SCCOHT sufficient to impair SWI/SNF-mediated tumor suppression.

Beyond BRG1, mutations to other subunits can disrupt tumor-suppressive SWI/SNF functions. For example, the gene encoding the SNF5 subunit of SWI/SNF is mutated in malignant rhabdoid tumors (MRTs) and atypical teratoid rhabdoid tumors [13]. SNF5 functions similarly to BRG1 in that, when expressed, it maintains SWI/SNF complex identity and activates enhancers linked to development genes [14,15,16,17,18]. These data, along with that generated in other SWI/SNF-altered cancers, has solidified the notion that proper SWI/SNF complex composition is necessary for the expression of genes that act in tumor suppression. What is less understood, however, is how SWI/SNF intersects with established oncogenic pathways as a means to control cancer formation, which is a newly emerging mechanism of tumorigenesis in these cancers [19]. We know that this connection likely involves interactions between SWI/SNF and oncogenic drivers but the extent to which these interactions occur and their significance across the diversity of SWI/SNF-altered cancers is unknown.

Recently, we proposed that a common mechanistic theme underlying the etiology of SWI/SNF-altered cancers involves interactions between SWI/SNF and oncoprotein transcription factors, one of them being MYC [19]. *MYC* oncogenes are involved in the majority of cancers, where *MYC* family members are either overexpressed or functionally deregulated [19]. The oncogenic power of MYC stems from its ability to control the expression of genes associated with diverse cellular processes that, when improperly constrained, promote cancer initiation, progression, and maintenance [20]. In MRT and other SNF5-deficient cancers, the activation of MYC target gene signatures is observed [21,22], which is thought to be due to the loss of SNF5-mediated inhibition of MYC functionality [19]. The activation of MYC target gene signatures is also present in SCCOHT tumors [23], and in BRG1-null lung cancer cell lines, the reintroduction of BRG1 antagonizes MYC function [24], pointing to a connection between BRG1 and MYC that may resemble that of SNF5. Currently, however, our understanding of how MYC features in SWI/SNF-altered cancers has been limited to SNF5-null cancer cells. We do not yet know the totality of genes regulated by MYC in cancers such as SCCOHT, nor do we understand the influence that SWI/SNF has on MYC activities in this context. Furthermore, interrogating the relationship between BRG1 and MYC in SCCOHT is a critical first step to comprehending the implications of recurrent MYC target gene expression observed in multiple SWI/SNF-altered cancers.

Here, we investigate the contribution of MYC to the biology of SCCOHT using genetically engineered cell lines. We find that MYC facilitates the expression of genes that either directly control DNA repair or indirectly regulate genomic stability. Using tumor expression data, we also discover that DNA repair gene expression is notably upregulated in SCCOHT and SNF5-deficient rhabdoid cancers. These data shed light on the relationship between MYC and BRG1 and implicate MYC as a key regulator of DNA repair gene expression in two SWI/SNF-altered cancers.

## 2. Materials and Methods

### 2.1. Cell Lines and Genetic Engineering

The BIN-67 cells were a gift from Dr. Bernard Weissman at the University of North Carolina Chapel Hill (Chapel Hill, NC, USA) and were maintained in RPMI-1640 with l-glutamine (Corning, Corning, NY, USA) containing 10% fetal bovine serum (Genesee Scientific, El Cajon, CA, USA) and 1% penicillin/streptomycin (Gibco, Jenks, OK, USA). Engineered G401 cells were generated previously [25] using parental G401 cells and HEK293 cells from ATCC (Manassas, VA, USA). These two cell lines were maintained in high-glucose DMEM (Corning) containing 10% fetal bovine serum and 1% penicillin/streptomycin. The cell lines used or generated in this study were confirmed by STR profiling (ATCC) and were regularly assessed for mycoplasma contamination using the Mycoplasma PCR Detection Kit (MP Biomedicals, Solon, OH, USA). To engineer BIN-67 cells to express c-MYC containing a N-terminal HA-FKBP12^F36V^ dTAG module, the lentiviral transfer plasmid generated previously [25] was transfected with psPAX2 and pMD2.G into HEK293 cells to produce a competent virus. Viral particles were collected in BIN-67 maintenance media and then used to transduce BIN-67 cells. The cells were transduced with 1 mL of viral supernatant once per day for two days and then allowed to recover in maintenance media before selection was performed by the addition of 1 μg/mL puromycin. Endogenous MYC expression was removed in the selected cells using CRISPR-Cas9 technology. For this, oligos containing a guide sequence against MYC (GCCGTATTTCTACTGCGACG) were annealed together, phosphorylated, and then inserted into the pSpCas9(BB)-2A-Blast using the BbsI sites. Plasmid sequences were confirmed by submission to MCLAB (San Francisco, CA, USA) and then the plasmid was used to transfect BIN-67 cells expressing the MYC-dTAG module using Lipofectamine 3000 (Invitrogen, Carlsbad, CA, USA). After three days, 10 μg/mL blasticidin was added to select for cells that received the plasmid. Once selected, the cells recovered and plated for single clones using a limited dilution into a 96-well plate. All clones recovered were tested for the presence of c-MYC with the dTAG module and the absence of endogenous c-MYC using parental cells as controls. Various plasmids were obtained from Addgene (Watertown, MA, USA). psPAX2 was a gift from Didier Trono (Addgene plasmid # 12260; https://www.addgene.org/12260/(accessed on 5 May 2025); RRID:Addgene_12260), pMD2.G was a gift from Didier Trono (Addgene plasmid # 12259; https://www.addgene.org/12259/(accessed on 5 May 2025); RRID:Addgene_12259), and pSpCas9(BB)-2A-Blast was a gift from Ken-Ichi Takemaru (Addgene plasmid # 118055; https://www.addgene.org/118055/(accessed on 5 May 2025); RRID:Addgene_118055).

### 2.2. Western Blots

Approximately 2.0 × 10^6^ cells were treated with either dimethyl sulfoxide (DMSO), 500 nM dTAG^V^-1-NEG (Tocris, 6915, Minneapolis, MN, USA), or 500 nM dTAG^V^-1 (Tocris, 6914) for 24 h before the cells were washed twice with phosphate-buffered saline (PBS), containing 137 mM NaCl, 2.7 mM KCl, and 10 mM phosphate (VWR, 76371-734, Radnor, PA, USA) and pelleted. Cell pellets were lysed in ice-cold lysis buffer that contained 150 mM Tris, pH 8.0, 1% Triton X-100, 5 mM EDTA, and 150 mM NaCl. The lysis buffer was supplemented with 0.001 M PMSF and a protease inhibitor cocktail. Once in the lysis buffer, the pellets were sonicated for 15 s at 25% power and then the soluble proteins were separated by centrifugation for 10 min at 13,000 RPM in a chilled centrifuge. Protein lysates were normalized to each other using the BioRad Bradford assay (BioRad, Hercules, CA, USA) to determine concentrations. For each Western blot, 10–25 μg of protein per sample was separated using SDS-PAGE, transferred to a PVDF membrane (Pierce, Rockford, IL, USA) and blocked in a 5% milk solution made in TBS (150 mM NaCl, 50 mM Tris, ph. 7.6) supplemented with 0.1% Tween-20 to make TBS-T. Primary antibodies were allowed to incubate overnight at 4°C. The next day, membranes were washed three times in TBS-T; secondary antibodies conjugated to horse radish peroxidase were added for 1 h at room temperature; and then, the membranes were washed an additional three times in TBS-T. For visualization, Clarity ECL substrate (BioRad) was added to the membranes for five minutes and bands were detected using a Bio-Rad Chemidoc MP instrument. The antibodies used for the Westerns include the following: MYC (Abcam, Cambridge, UK, AB32072), BAF155 (Cell Signaling, 11956, Waltham, MA, USA), SNF5 (Cell Signaling, 91735), ATR (Cell Signaling, 13934), RAD51 (Cell Signaling, 8875), and GAPDH-HRP (Cell Signaling, 8884).

### 2.3. Cell Proliferation and Cell Cycle Analysis

For proliferation experiments, 5.0 × 10^5^ cells were plated with DMSO or 500 nM dTAG^V^-1-NEG and 1.0 × 10^6^ cells were plated with 500 nM dTAG^V^-1. All cells were placed back into the tissue culture incubator for five days. At the end point, cells from each sample were counted using an Invitrogen Countess II automated cell counter. Total cell counts on day five were determined by multiplying the cell count in cells per ml by the volume resuspended. Fold change over the course of five days was calculated by dividing the total cell count on day five by the number of cells plated. A cell cycle analysis was performed similarly except at day five 1.0 × 10^6^ cells were harvested in 500 μL PBS and slowly added dropwise to 4.5 mL of ice-cold 70% ethanol while vortexing at low speed. Fixed cells were stored at −20°C for at least 24 h before proceeding to staining. For staining, tubes were thawed for a minimum of 30 min at room temperature, and then, the cells were pelleted at 800× *g* for 6 min. The cell pellets were gently washed in PBS, pelleted again, and then resuspended in a staining solution (PBS, 100 μg/mL RNAse A inhibitor, 2 mM MgCl2) containing 10 μg/mL propidium iodide. The samples were allowed to stain overnight at 4°C before filtering through a 35 μm nylon mesh strainer. A minimum of 10,000 events were recorded per sample using a Guava easyCyte Flow Cytometer (Luminex, Austin, TX, USA). Forward and side scatter was used to select single cells.

### 2.4. CUT&RUN

CUT&RUN was performed by employing the CUT&RUN Assay Kit (Cell Signaling, 72917 and 91931). In brief, 500,000 engineered BIN-67 cells were collected per reaction in 1X Wash Buffer provided in the kit according to the manufacturer’s instructions. The cells in each reaction tube were bound to concanavalin A (ConA) beads and incubated overnight at 4°C on a rotator with the antibodies. The antibodies used in this study were a MYC antibody (Cell Signaling, 13987, 2 μL per reaction), a H3K4me3 antibody (included in the kit, 2 μL per reaction), or normal IgG (Cell Signaling, 2729, 5 μL per reaction) as a negative control. Following overnight incubation, all samples were resuspended in a pAG-MNase mixture and left on the rotator for 1 h at 4°C. The pAG-MNase enzyme was activated in each reaction tube by the addition of the calcium chloride solution included in the kit, which was allowed to occur at 4°C for 30 min. Then, a 1X Stop Solution was added to end the reaction and all samples were incubated for 10 min at 37°C to release the DNA fragments before centrifugation for 2 min at high speeds to separate out the free DNA. DNA was then purified for library preparation using the Cell Signaling DNA purification kit (14209). For IgG and MYC, the equivalent of three reactions was pooled during DNA purification per replicate. For H3K4me3, one reaction was sufficient for library generation. The NEBNext Ultra II DNA library prep kit for Illumina (New England Biolabs, E7645, Ipswich, MA, USA) and NEBNext multiplex oligos for Illumina (E6440S) were used. Libraries were sequenced with 150 pair end reads on a NovaSeq6000 at the Vanderbilt Technologies for Advanced Genomics core located at the Vanderbilt University Medical Center. Three biological replicates were performed for CUT&RUN analysis.

### 2.5. mRNA Analysis and RNA-Seq

Approximately 2.0 × 10^6^ engineered cells were plated with DMSO, 500 nM dTAG^V^-1-NEG, or 500 nM dTAG^V^-1 for 24 h, and then, the cells were harvested into 600 μL of Trizol (Invitrogen). RNA was purified using the Direct-zol RNA mini-prep kit (Zymo Research, Tustin, CA, USA). For RNA-seq, 2 μg was sent to Novogene (Sacramento, CA, USA), who handled all downstream poly(A) enrichment, library generation, and 150 bp paired end sequencing on a NovaSeq6000. Three biological replicates were performed for RNA-seq. For mRNA analysis, purified RNA was reverse transcribed using MulV reverse transcriptase (Promega, Madison, WI, USA) and the levels of expression were quantified using an AriaMx Real-Time PCR Machine (Agilent, Santa Clara, CA, USA), with *GAPDH* included as a reference gene for normalization. The primers used in this study are as follows: RSP24_fwd: 5′-GACACCGTAACTATCCGCACT-3′, RPS24_rev: 5′-TCTTAGGCACTGTCGCCTTC-3′, RNPS1_fwd: 5′-GACAAAACCCGAAAGAGG-3′, RNSP1_rev: 5′-GAGCCTGAGCTGGAAGTC-3′, SNHG15_fwd: 5′-TCTCAAAGTGGAAGAGCTTACTG-3′, SNHG15_rev: 5′-GACTCAAGGAGGGACCTCAG-3′, PUMA_fwd: 5′-CTCAACGCACAGTACGAG-3′, PUMA_rev: 5′-GAGTCCCATGATGAGATTGT-3′, RPL35_fwd: 5′-AACAGCTGGACGACCTGAAG-3′, RPL35_rev: 5′-ACTGTGAGAACACGGGCAAT-3′, CCT7_fwd: 5′-AAGCTTATTGTAGATGGCAGA-3′, CCT7_rev: 5′-GGATGGACAACATCAAGAAG-3′, GAPDH_fwd: 5′-AAGGTGAAGGTCGGAGTCAAC-3′, GAPDH_rev: 5′-GTTGAGGTCAATGAAGGGGTC-3′.

### 2.6. CUT&RUN and Chromatin Binding Analyses

For MYC and H3K4me3 chromatin binding, CUT&RUNTools [26] was used for all aspects of analysis, including trimming adapters, alignment to the hg38 genome, duplication marking, and peak calling. DiffBind was used to obtain consensus peaks for MYC and H3K4me3 [27], and annotation of the peaks was performed using the HOMER program [28] and ‘annotatePeaks.pl’. The binding sites for BAF155 in BIN-67 cells, and BAF155 and K785R BRG1 in BIN-67 cells with reintroduced K785R BRG1 were obtained from the ChIP-seq data deposited under GSE117734 [15]. This entailed downloading the deposited SCCOHT raw peaks identified in that study (two replicates for BAF155_Control_ChIPseq, with peak calling against the Input_Control_ChIPseq, and one replicate each for BAF155_SMARCA4_K785R_ChIPseq and BRG1_SMARCA4_K785R_ChIPseq, with peak calling against Input_SMARCA4_K785R_ChIPseq) and using the LiftOver tool to align the peaks to the hg38 genome. The peaks aligned to the hg38 genome were used to analyze peak overlap with MYC CUT&RUN peaks.

### 2.7. RNA-Seq Analysis

Adapters were trimmed using Cutadapt [29] with the following parameters: cutadapt-j2-q20-aAGATCGGAAGAGCACACGTC-AAGATCGGAAGAGCGTCGTGT-m30-n3--trim-n. The reads were aligned to the hg38 genome using STAR [30], and the reads were quantified using featureCounts [31]. DEseq2 [32] was used to perform a normalization and differential analysis for the BIN-67 RNA-seq experiments generated in this study. The Benjamini–Hochberg procedure was used to determine Wald test *p*-values, log2 fold changes, and adjusted *p*-value (false discovery rate). Genes that were significantly changed were called using a false discovery rate < 0.05.

### 2.8. Expression Analysis in Tumor Samples

For the SCCOHT analysis, the RNA expression data for 10 SCCOHT tumors was analyzed as described previously [14] and is located under dbGaP: phs001528.v1.p1, GSE109919, and GSE216801. In brief, SCCOHT tumors that were validated for the absence of SMARCA4/BRG1 and SMARCA2/BRM expression, as determined by immunohistochemistry, were processed for RNA extraction and submitted for next-generation sequencing. FPKM values were calculated for each dataset using TGen’s Phoenix pipeline [14], which normalizes gene counts based on gene length. Batch correction was not used. FPKM data for four normal ovary tissues were obtained from the Encyclopedia Of DNA Elements (ENCODE; ENCODE ovary). FPKM data for 45 HGSC cancers were obtained from The Cancer Genome Atlas (TCGA) and included patients under 50 years of age to achieve closer age-matching with the SCCOHT patients. FPKM + 0.01 values (FPKMadj) were used for all comparisons to obtain an expression value greater than zero for all genes analyzed. For the rhabdoid tumor analysis, normalized RNA-seq count values for 55 primary rhabdoid tumor samples and six normal kidney samples were downloaded from the NCI Therapeutically Applicable Research to Generate Effective Treatments (TARGET) initiative (http://ocg.cancer.gov/programs/target, accessed on 12 December 2023) [33]. The differential expression between the tumor and normal samples was assessed using a *t*-test for each gene.

## 3. Results

### 3.1. Depletion of MYC Impairs SCCOHT Cell Line Function

**Figure 1:**
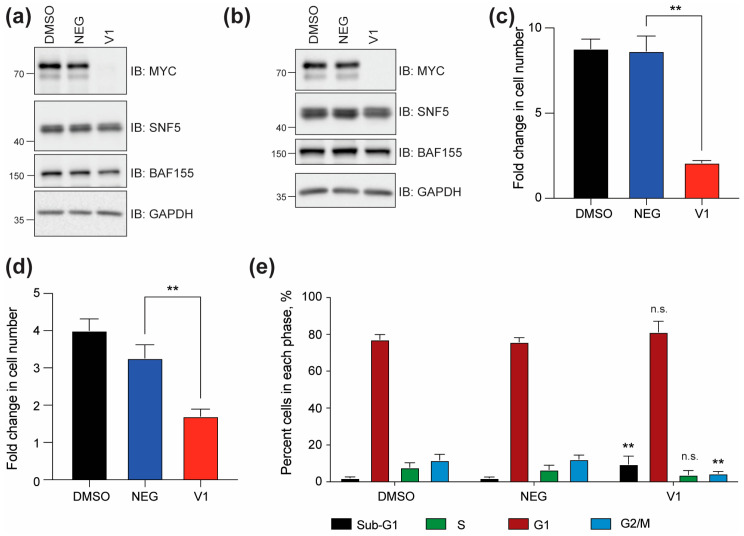
Depletion of MYC in BIN-67 cells. (**a**) Engineered BIN-67 (clone 1) cells were treated with DMSO, 500 nM dTAG^V^-1-NEG (NEG), or 500 nM dTAG^V^-1 (V1) for 24 h to deplete MYC. GAPDH is included as a loading control. SNF5 and BAF155 are SWI/SNF subunits. Uncropped Western blots are included in Figure S1. (**b**) Engineered BIN-67 (clone 9) cells were treated as in (**a**) for 24 h to deplete MYC. Similar controls are included. Uncropped Western blots are included in Figure S1. (**c**) Engineered BIN-67 (clone 1) cells were plated with 500 nM NEG, 500 nM V1, or DMSO and then allowed to grow for five days before recounting. The fold change in cell number was determined using the total cell number obtained on day five as compared to the day of plating. (The error bars are the standard error of the mean, unpaired student’s *t*-test ** *p*= 0.0019, *n* = 3 biological replicates). (**d**) The engineered BIN-67 (clone 9) cells were plated as in (**c**), and the fold change was calculated and graphed. (The error bars are the standard error of the mean, unpaired student *t*-test ** *p* = 0.0089, *n* = 4 biological replicates). (**e**) The engineered BIN-67 (clone 1) cells were plated with 500 nM NEG, 500 nM V1, or DMSO. After five days, a cell cycle analysis was performed, and the data were quantified for each phase, as shown. (The error bars are the standard error of the mean, one-way ANOVA analysis Sub-G1 ** *p* = 0.0030, G2/M ** *p* = 0.0018, n.s. = not significant, *n* = 4 biological replicates).

To examine the contribution of MYC to SCCOHT, we engineered the SCCOHT cell line BIN-67 to express the c-MYC family member with an in-frame N-terminal HA-FKBP12^F36V^ dTAG module and then eliminated endogenous c-MYC expression using CRISPR-Cas9 technology. In multiple BIN-67 clonal cell lines, the addition of a dTAG^V^-1 molecule (“V1”) [34] induced complete depletion of MYC in 24 h as compare to treatment with DMSO or the heterobifunctional negative control, dTAG^V^-1-NEG (“NEG”) (Figure 1a,b). The levels of the retained SWI/SNF subunits SNF5 and BAF155 were not overtly affected by V1 treatment. The depletion of MYC in either clone caused significant impairment in cell proliferation when compared to the treatment with the negative control (Figure 1c,d). Because the degree of MYC degradation and response to the depletion of MYC were similar among both clones, we proceeded with our downstream analysis using BIN-67 (clone 1). Using this cell line, we confirmed that treatment with V1 also impacts cell function by decreasing the number of cells in the G2/M phase of the cell cycle and increasing the number of cells in sub-G1 (Figure 1e), which is indicative of a depletion of MYC causing cell death.

### 3.2. Characterization of MYC Binding Sites in BIN-67 Cells

**Figure 2:**
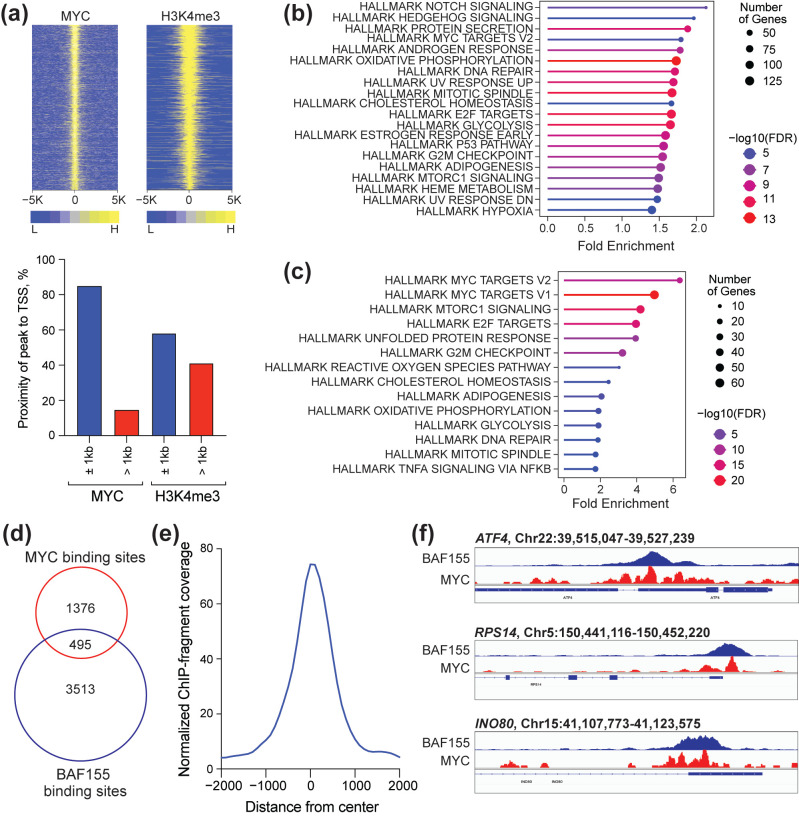
Characterization of MYC chromatin occupancy in BIN-67 cells. (**a**) Top: Heatmap of normalized MYC and H3K4me3 peak intensity, as determined by CUT&RUN. Signal for each protein within 5 kb from center of peaks is shown in yellow. Bottom: Identified peaks were annotated to their nearest gene, and proximity of peaks to the TSS is shown. (**b**) Peaks with H3K4me3 signal within 1 kb of a TSS were annotated to their nearest gene and analyzed for enrichment among Hallmark.MSigDB gene sets using ShinyGO 0.82 [36]. (**c**) Peaks with MYC signal within 1 kb of a TSS were annotated to their nearest gene and analyzed for enrichment among Hallmark.MSigDB gene sets using ShinyGO 0.82. (**d**) Venn diagram showing the overlap between the number of MYC binding sites detected in this study with those of BAF155 in BIN-67 cells using publicly available sequencing data (GSE117734, [15]). (**e**) Averaged ChIP-seq fragment coverage for BAF155 at MYC-BAF155 colocalized regions. (**f**) Binding of MYC and BAF155 at three MYC-BAF155 cobound genes. Image created on Integrative Genomics Viewer [37] using chromatin binding data for both proteins.

To dissect the contribution of MYC to gene expression in SCCOHT, we began by performing a genome-wide chromatin binding analysis using Cleavage Under Targets and Release Using Nuclease (CUT&RUN) coupled to next-generation sequencing in the engineered BIN-67 cells. We included antibodies against MYC as well as the histone mark histone 3 lysine 4 trimethylation (H3K4me3) to identify active promoters [35]. We detected ~1800 MYC peaks and ~17,000 H3K4me3 peaks, with 99% of MYC peaks overlapping with a H3K4me3 peak. Approximately 80% of MYC peaks are localized within 1 kb of a transcription start site (TSS), compared to ~60% for H3K4me3 (Figure 2a). To examine the types of genes with active promoters, we annotated H3K4me3 peaks located within 1 kb of a TSS to their nearest gene and analyzed the genes for enrichment among Hallmark.MSigDB gene sets using ShinyGO 0.82 [36]. We find that these genes are clustered within gene sets related to signaling, metabolism, DNA repair, cell cycle, and MYC (Figure 2b). We performed the same analysis for genes having an MYC signal within 1 kb of a TSS and extracted many of the same enriched gene categories, showing “MYC targets V2” and “MYC targets V1” as gene sets with the highest enrichment scores (Figure 2c).

Because retained SWI/SNF complexes promote aberrant genomic activities in SWI/SNF-altered cancers—some of which occurs through interactions with MYC on chromatin [19]—we also analyzed chromatin immunoprecipitation sequencing (ChIP-seq) data that are publicly available for the SWI/SNF subunit BAF155 in BIN-67 cells [15]. We compared the number of binding sites detected for MYC to those determined for BAF155 and observe that only one third of MYC binding sites show colocalization with BAF155 (Figure 2d). The BAF155 signal at MYC-BAF155 colocalized sites is high (Figure 2e) and colocalization occurs at important genes involved in chromatin regulation and protein synthesis (Figure 2f). However, based on the small degree of colocalization between MYC and BAF155, this analysis suggests that retained SWI/SNF subunits may not profoundly contribute to MYC-dependent gene regulation in this cancer context.

### 3.3. Depletion of MYC Impairs Expression of a Broad Spectrum of Genes

**Figure 3:**
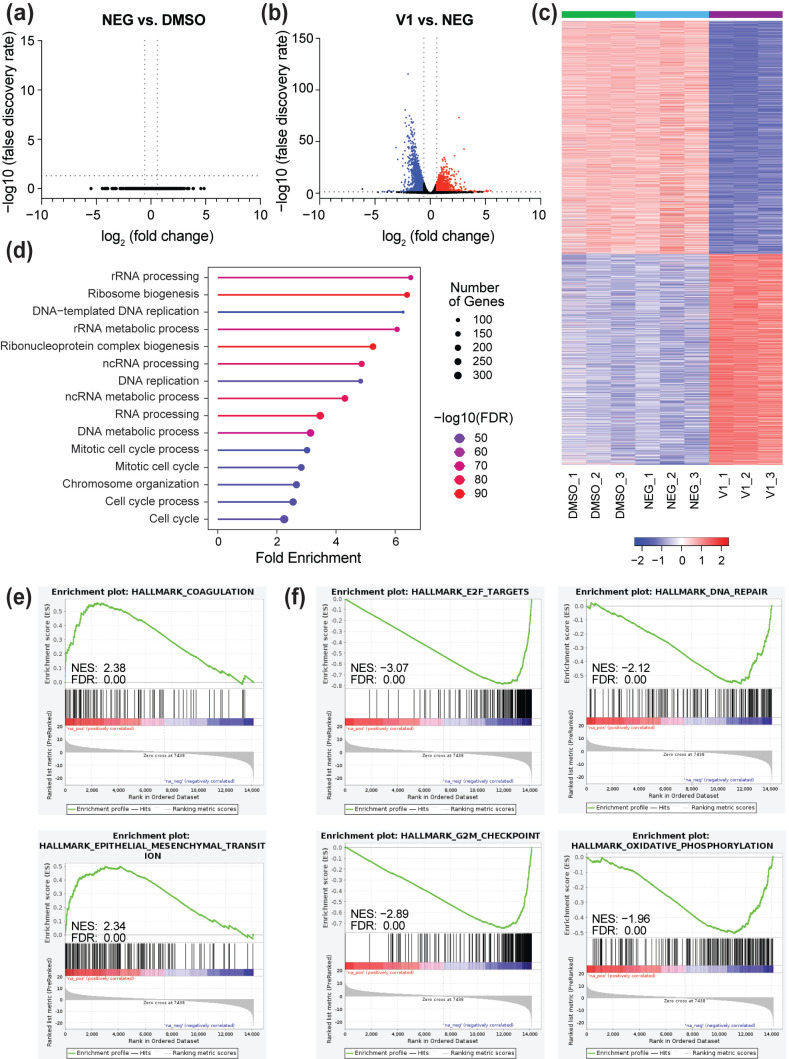
Depletion of MYC impacts the expression of diverse sets of genes. (**a**) The magnitude and significance of gene expression changes resulting from an RNA-seq analysis of engineered BIN-67 cells treated with DMSO or 500 nM dTAG^V^-1-NEG (NEG) for 24 h. The grey lines denote a false discovery rate (FDR) of 0.05 and a fold change of 1.5. (**b**) A volcano plot showing the magnitude and significance of gene expression changes from an RNA-seq analysis of engineered BIN-67 cells treated with 500 nM NEG or dTAG^V^-1 (V1). The blue dots indicate genes that decrease significantly in expression (FDR < 0.05) with a fold change less than −1.5. The red dots indicate genes that increase significantly (FDR < 0.05) in expression and have a fold change that is higher than 1.5. (**c**) A heatmap depicting z-score-normalized read counts for genes that significantly changed in expression (FDR < 0.05) following treatment with V1. The data for each replicate is included. (**d**) A gene ontology analysis was performed using genes that decrease in expression following MYC depletion, as determined in the V1 vs. NEG analysis (FDR < 0.05, fold change < −1.5). The analysis was performed using ShinyGO 0.82 [36] and “GO Biological Process”. (**e**) A gene set enrichment analysis (GSEA) was performed by testing the differentially expressed genes (V1 vs. NEG) against MSigDB hallmark datasets [38,39]. The loss of MYC causes an upregulation of genes related to coagulation and epithelial mesenchymal transition. (**f**) GSEA was performed as in (**e**). The loss of MYC downregulates genes related to E2F targets, DNA repair, G2/M checkpoint, and oxidative phosphorylation. The complete GSEA results are in Table S2.

Next, we performed RNA-seq on engineered BIN-67 cells following 24 h of treatment with DMSO, 500 nM NEG, or 500 nM V1, a timepoint in which MYC is fully depleted (Figure 1a). An RNA-seq analysis of the NEG-treated samples versus the DMSO-treated samples shows no significant gene expression changes (Figure 3a), which is consistent with the inability of the negative control compound to induce protein degradation [34]. In contrast, an analysis of the V1-treated samples versus the NEG-treated samples results in thousands of significant gene expression changes (Figure 3b), with ~2900 genes increasing in expression and ~3200 genes decreasing in expression following the depletion of MYC (Figure 3c and Table S1). Using a gene ontology analysis, we find that genes suppressed by V1-treatment (false discovery rate (FDR) < 0.05, fold change < −1.5) are enriched among biological processes related to cell cycle, ribosome biogenesis, and nucleic acid processing (Figure 3d), some of which we validated with a targeted mRNA analysis (Figure S2a). In contrast, genes that increase in expression upon MYC depletion (FDR < 0.05, fold change > 1.5) are enriched among gene categories broadly related to development and differentiation (Figure S2b).

Because of the sheer number of gene expression changes induced by MYC, we also analyzed the V1 vs. NEG RNA-seq data using a gene set enrichment analysis (GSEA) [38,39] and a KEGG pathway analysis [40] to extract additional information on the types of genes regulated by MYC. The GSEA analysis against MSigDB Hallmark datasets shows that genes that increase in expression are enriched within many distinct and informative gene sets including coagulation and epithelial mesenchymal transition (Figure 3e, Table S2), while genes that decrease in expression are also significantly enriched among many hallmark sets, including those related to E2F targets, DNA repair, and oxidative phosphorylation (Figure 3f and Table S2). Strikingly, when we perform a KEGG pathway analysis on suppressed genes (FDR < 0.05, fold change < −1.5) using ShinyGO 0.82 [36], we see a notable emphasis on genes related to DNA repair pathways (Figure S2c) that are distinct from genes involved in ribosome biogenesis or cell cycle (Figure S2d). Taken together, we conclude that MYC plays an instrumental role in sculpting the gene expression program in BIN-67 cells and that the expression of diverse sets of genes are influenced by the loss of MYC.

### 3.4. Regulation of DNA Repair Gene Expression by MYC and BRG1

#### 3.4.1. MYC Facilitates Expression of DNA Repair Genes

**Figure 4:**
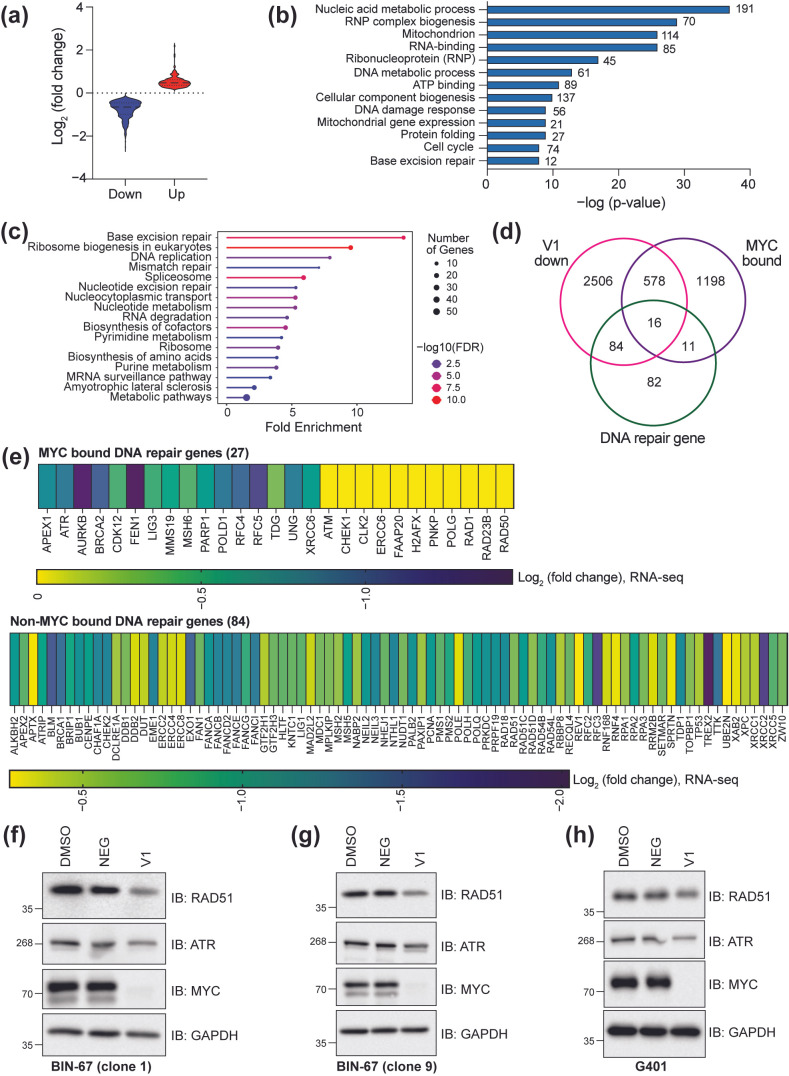
Gene regulation by chromatin-bound MYC. (**a**) Magnitude of change in expression for genes bound by MYC that go up (red) or down (blue) following depletion of MYC in BIN-67 cells. MYC-bound genes are defined as a gene with an MYC binding site localized within 1 kb of the annotated TSS. (**b**) Gene ontology analysis of MYC-bound genes that decrease in expression following the depletion of MYC using DAVID Bioinformatics [41,42]. The number next to each bar signifies the number of genes in that category. (**c**) A KEGG pathway analysis was performed on MYC-bound genes that decrease in expression following MYC depletion. An analysis was performed using ShinyGO 0.82 [36] and the “KEGG pathway database” [40]. (**d**) A Venn diagram comparing the number of significantly downregulated genes in the V1 vs. NEG RNA-seq analysis with genes annotated to MYC binding sites and a curated list of DNA repair genes from [43]. The Venn diagram was generated using https://molbiotools.com/listcompare.php(Accessed on 3 March 2025). (**e**) Top: heatmap showing the magnitude of gene expression changes for the 27 MYC-bound DNA repair genes from (**d**). Genes marked with solid yellow are bound by MYC but have no detectable change in gene expression. Bottom: heatmap showing the magnitude of gene expression changes for the 84 non-MYC-bound DNA repair genes from (**d**). (**f**) Western blot showing the levels of MYC, ATR, and RAD51 in BIN-67 (clone 1) cells treated with DMSO, 500 nM NEG, or 500 nM V1 for 24 h. GAPDH serves as a loading control. (**g**) Western blot as described in (**f**) for BIN-67 (clone 9) cells treated similarly. (**h**) Western blot as described in (**f**) for engineered SNF5-null G401 cells treated similarly. Uncropped Western blots are included in Figure S1.

To know which genes are directly controlled by MYC, we compared the genes with an annotated MYC binding site, as determined by CUT&RUN (Figure 2), with the differentially expressed genes detected in the V1 vs. NEG RNA-seq analysis (Figure 3). Almost half of genes with an MYC binding site show changes in expression at this timepoint (deemed as “MYC targets”), with the magnitude of changes for suppressed genes being larger than those that increase in expression (Figure 4a). The gene ontology analysis performed for MYC targets that decrease in expression using DAVID Bioinformatics [41,42] shows that MYC directly regulates genes involved in many of the functions we already identified (Figure 4b). Curiously, however, a KEGG pathway analysis of these same genes again highlights DNA replication and DNA repair pathways as enriched pathways for suppressed MYC targets (Figure 4c). Because of the apparent impact of MYC depletion on genes associated with DNA repair—and the observation that MYC localizes to promoters of DNA repair genes—we compared our data to a comprehensive list of 193 genes that directly contribute to DNA repair or indirectly influence genomic stability [43]. In doing so, we observe that half of the 193 DNA repair genes analyzed are suppressed upon loss of MYC, some of which are also bound by MYC (Figure 4d). In total, 27 DNA repair genes from this list are MYC targets and include many well-characterized genes such as *ATR*, *ATM*, *BRCA2*, and *PARP1*, while an additional 84 DNA repair genes show no detectable MYC binding but respond to a depletion of MYC (Figure 4e). We determined whether these changes correspond with a change in protein level by examining two MYC-responsive genes: *ATR*, a direct MYC target, and *RAD51*, a non-MYC-bound target. Here, we observe that the levels of both proteins are decreased following the depletion of MYC (Figure 4f), an effect that holds true in the second engineered BIN-67 clonal cell line (Figure 4g). We also performed a degradation of MYC in the SNF5-null MRT cell line, G401, which we previously engineered to investigate the influence of MYC on MRT cell function [25]. In G401 cells, the depletion of MYC results in a similar decreased level of both ATR and RAD51 (Figure 4h), suggesting that MYC generally controls DNA repair protein expression in SWI/SNF-altered cancer cell lines, as has been seen in other cancers [44,45,46].

#### 3.4.2. BRG1 Antagonizes Expression of DNA Repair Genes

**Figure 5:**
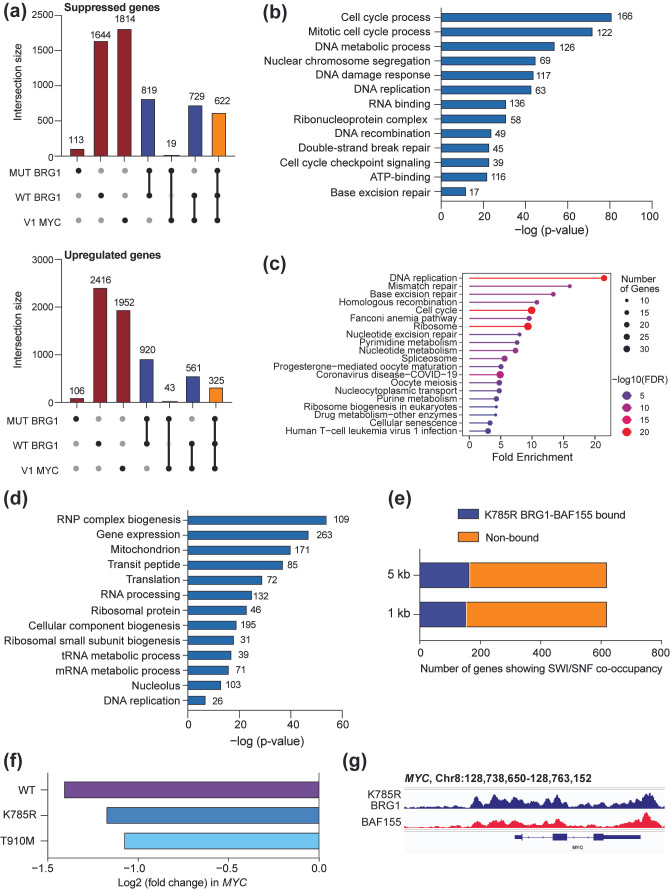
Gene regulation by MYC and BRG1. (**a**) UpSet plot showing the intersection of genes that significantly decrease following the depletion of MYC in BIN-67 cells (V1 MYC) (top) or increase (bottom) with genes that significantly change in the same direction following the reintroduction of wild-type BRG1 (WT BRG1) or ATPase-deficient T910M and K785R BRG1 (MUT BRG1). Gene expression changes following BRG1 reintroduction were reported in [15]. Black dots indicate the gene set(s) included in the intersection analysis and lines indicate the overlap of multiple sets. (**b**) Gene ontology analysis of the 622 commonly suppressed genes from (**a**) using DAVID Bioinformatics [41,42]. The number next to each bar signifies the number of genes in that category. (**c**) A KEGG pathway analysis was performed on the 622 commonly suppressed genes from (**a**). An analysis was performed using ShinyGO 0.82 and the “KEGG pathway database”. (**d**) A gene ontology analysis of the 729 genes suppressed by MYC depletion and wild-type BRG1 reintroduction from (**a**) using DAVID Bioinformatics [41,42]. The number next to each bar signifies the number of genes in that category. (**e**) Stacked bar graph showing the number of commonly regulated genes from (**a**) that show chromatin binding of ATPase-deficient BRG1 (K785R) and BAF155 within either 1 kb or 5 kb of a TSS. Chromatin binding data for BAF155 and K785R BRG1 in BIN-67 cells was obtained using publicly available data in GSE117734 [15]. (**f**) Log2(fold change) in *MYC* expression as analyzed in [15] when wild-type (WT) or ATPase-deficient T910M BRG1 and K785R BRG1 are reintroduced in BIN-67 cells. (**g**) K785R BRG1 and BAF155 ChIP-seq peaks at the *MYC* gene in BIN-67 cells following the reintroduction of K785R BRG1. Image created on Integrative Genomics Viewer [37] using chromatin binding data for both proteins.

If MYC facilitates the expression of genes used in DNA repair as part of its oncogenic function in SCCOHT, then we theorized that BRG1 would act to oppose the expression of MYC-regulated genes. To test this hypothesis, we compared the genes that change in expression following MYC depletion to genes previously reported to significantly change in expression following the reintroduction of BRG1 in BIN-67 cells [15]. Because BRG1 retargets ~25% of SWI/SNF complexes in a catalytic-independent manner and not all transcriptional changes can be accounted for by the ATPase function of BRG1 [11], we also included a comparison of genes changed by the reintroduction of BRG1 that lacks full ATPase activity. For this, we generated a list of high-confidence genes regulated by ATPase-deficient BRG1 by identifying genes that increased or decreased significantly in expression following the reintroduction of BRG1 that has no catalytic activity (K785R BRG1) and reduced catalytic activity (T910M BRG1). As expected, the reintroduction of wild-type BRG1 results in a substantially higher number of gene expression changes than ATPase-deficient BRG1 (Figure 5a). We identified a group of 622 genes that decrease in expression in a catalytic activity-independent manner and are also downregulated by the loss of MYC, while 325 genes increase in expression. Notably, the 622 suppressed genes are heavily enriched among biological functions related to cell cycle, DNA repair, DNA replication, and DNA damage (Figure 5b,c). This is in contrast to the 729 genes suppressed by wild-type BRG1 and MYC (Figure 5a), which tend to cluster more within biological functions related to mRNA, mitochondrial function, and protein synthesis (Figure 5d). These results indicate that genes related to DNA repair are predominantly regulated by BRG1 in a catalytic activity-independent manner. To know if ATPase-deficient BRG1 regulates this group of genes directly, we analyzed publicly available ChIP-seq data for K785R BRG1 and BAF155 following the reintroduction of K785R BRG1 [15] to identify K785R BRG1-BAF155 cobound sites. We find that ~30% of the 622 commonly suppressed genes have detectable K785R BRG1-BAF155 cooccupancy within 5 kb (Figure 5e), suggesting that ATPase-deficient BRG1 does not control the majority of these genes directly. We then asked whether the effects we observed could be explained by ATPase-deficient BRG1 affecting *MYC* expression. In doing this, we find that the reintroduction of all three forms of BRG1 (wild-type, T910M, and K785R) reduce the expression of *MYC*, with much of the effect being independent of the ATPase function of BRG1 (Figure 5f). Both K785R BRG1 and BAF155 colocalize on chromatin at the *MYC* TSS and the transcription termination site following the reintroduction of K785R BRG1 (Figure 5g), suggesting that BRG1 may regulate *MYC* expression directly through a non-catalytic activity mechanism. These data reveal that MYC and BRG1 oppositely regulate the expression of genes related to DNA repair, with MYC generally facilitating expression and BRG1 tempering it.

### 3.5. Upregulation of DNA Repair Gene Expression in SCCOHT Tumors

**Figure 6:**
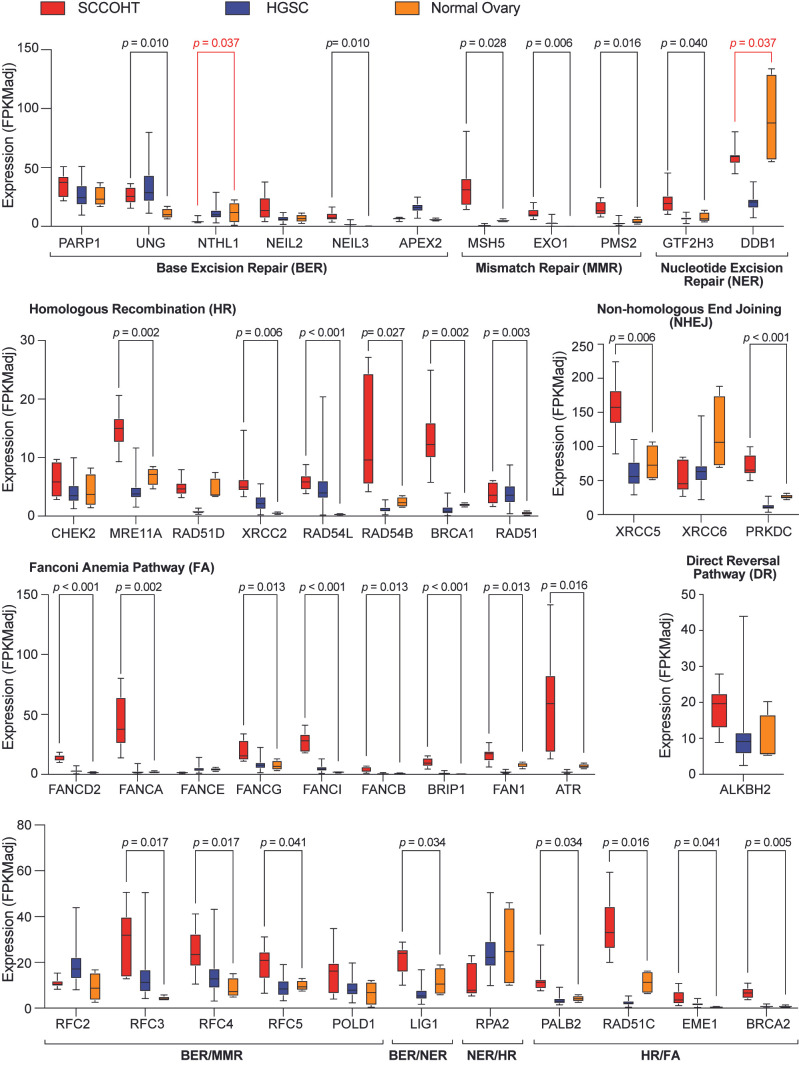
Expression of DNA repair genes is increased in SCCOHT tumors. RNA expression values in 4 normal ovary tissues, 10 SCCOHT tumors, or 45 age-matched high-grade serous ovarian carcinoma (HGSC) cancers for DNA repair genes regulated by MYC in BIN-67 cells. Genes were categorized based on their direct role within one or more specific repair pathways [43]. Box-and-whisker plots show expression of each gene within the indicated repair pathway(s). Boxes mark the 25th to 75th percentiles, with a middle line indicating the median value and whiskers extending from highest to lowest data points. Student’s unpaired *t*-test was performed on data from SCCOHT tumors and normal ovary tissue for each gene, and *p*-values are shown. Black *p*-values indicate a significant increase in SCCOHT tumors versus normal ovary tissue, and red *p*-values indicate a significant increase in normal ovary tissue versus SCCOHT tumors. A statistical analysis was also performed on data from SCCOHT tumors versus HGSC cancers, and those *p*-values are located in Table S3.

Dysregulated DNA repair gene expression contributes to multiple facets of cancer, including initiation, progression, and metastasis [47,48]. Furthermore, the upregulation of these types of genes can affect the response of tumors to chemotherapy and radiation [47] and can underly resistance to treatment [48]. To interrogate the significance of our cell-based findings in tumors, we sorted over 40 genes suppressed by MYC depletion in BIN-67 cells into one or more of the following direct DNA repair pathways: base excision repair, mismatch repair, nucleotide excision repair, homologous recombination, non-homologous end joining, Fanconi anemia pathway, and direct reversal pathway [43]. These pathways include DNA repair genes that we identified as MYC targets such as *UNG*, *XRCC6*, *ATR*, *RFC4*, *RFC5*, *POLD1*, and *BRCA2* (Figure 4e). Then, we examined the expression level of these genes in 10 SCCOHT tumors that were devoid of BRG1 and BRM expression, as determined by immunohistochemistry, to exclude any other tumors that commonly mimic this malignancy and were analyzed previously [14,49]. We compared the RNA expression values in these tumors to the expression values obtained from ENCODE for four normal ovary tissue (ENCODE) samples or from the TCGA for 45 tumors from age-matched HGSC cancers. For all pathways except direct reversal, the majority of genes analyzed were upregulated in SCCOHT as compared to normal ovary tissue (Figure 6). For the most part, the expression of these genes in SCCOHT tumors was upregulated as compared to HGSC cancers too, revealing a stark increase in DNA repair gene expression specific to SCCOHT (Table S3). To determine if the apparent effect on DNA repair gene expression is selective, we also sorted over 20 genes that are suppressed by MYC depletion in BIN-67 cells and are involved in oxidative phosphorylation (Figure 3f). This analysis reveals that oxidative phosphorylation gene expression in SCCOHT is not significantly upregulated compared to that of normal ovary tissue (Figure S3) and only somewhat upregulated compared to HGSC cancers (Table S3).

### 3.6. Upregulation of DNA Repair Gene Expression in Rhabdoid Tumors

**Figure 7:**
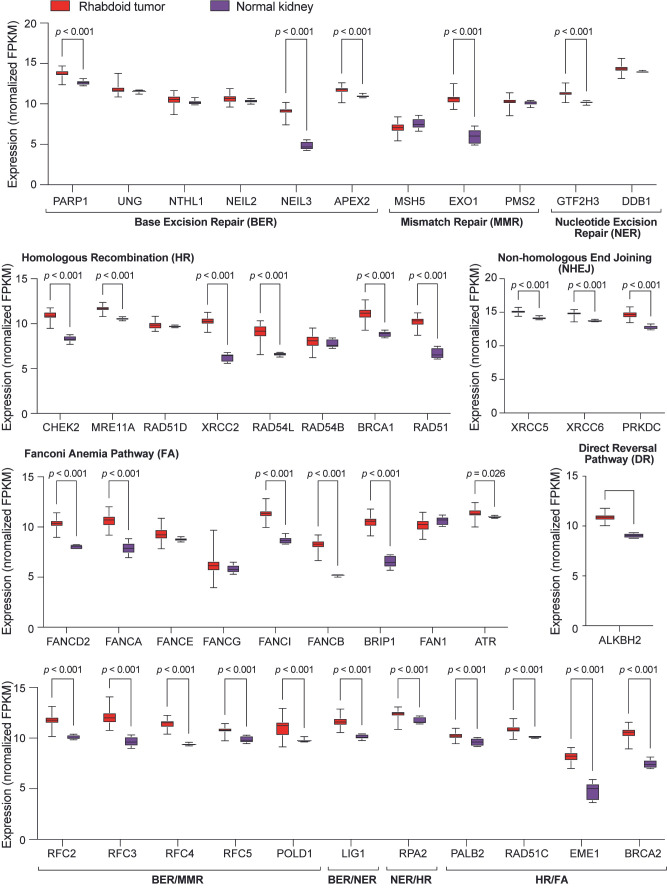
Expression of DNA repair genes is increased in rhabdoid tumors. RNA expression levels of the DNA repair genes shown in Figure 6 were analyzed in 55 primary rhabdoid tumors or 6 normal kidney tissues. Boxes show the 25th to 75th percentiles, with a middle line marking the median value and whiskers extending from highest to lowest data points. Student’s unpaired *t*-test was performed on data from rhabdoid tumors and normal kidney tissue for each gene, and significant *p*-values are shown.

Having identified an increased DNA repair gene signature in SCCOHT tumors that is likely related to MYC activity, we turned our attention to SNF5-deficient rhabdoid tumors because of the established connection between SNF5 and MYC in cell lines from these cancers [19]. To accomplish this, we extracted expression data for 55 rhabdoid tumors and 6 normal kidney tissues from the NCI Therapeutically Applicable Research to Generate Effective Treatments (TARGET) initiative [33] and compared the expression of these same genes. Remarkably, we uncover upregulation of more than 70% of the gene signature in rhabdoid tumor samples as compared to normal kidney (Figure 7), including *ALKBH2*, which contributes to the direct reversal pathway. This result suggests that the dysregulation of DNA repair gene expression is a general feature of more than one type of SWI/SNF-altered cancer.

## 4. Discussion

SCCOHT is a rare and aggressive ovarian cancer with dismal survival rates. While new and targeted therapies show efficacy in cell lines and mouse models [14,50], novel treatments have yet to be FDA-approved, leaving chemotherapy and extensive surgery as the predominant treatment approach [3]. From a mechanistic perspective, SCCOHT is the most well-characterized of BRG1-null cancers, although *SMARCA4* mutations also occur in up to 10% of non-small cell lung cancers sequenced [51]. As such, much of what we understand about the molecular underpinnings of BRG1-null cancers is inferred from studies in SCCOHT. In these studies, there is a clear role for BRG1 in the regulation of SWI/SNF complex identity and function that heavily influences enhancer and super-enhancer-mediated gene regulation [14,15,16]. However, if and how BRG1 intersects with known oncogenic pathways is unexplored territory yet, if understood, could inform how BRG1 operates to maintain such potent tumor suppression.

In this study, we explore how a major oncogenic driver with deep ties to cancer—MYC—influences the SCCOHT transcriptome. We find that MYC is localized at promoters of genes involved in biological functions related to cell growth and division, metabolism, DNA repair, and cell cycle (Figure 2). When we assessed whether retained SWI/SNF subunits colocalize with MYC on chromatin, we found that only one third of MYC binding sites are also occupied by retained BAF155 (Figure 2d–f). This is in contrast to SNF5-null MRT cell lines where ~70% of MYC colocalizes with retained BAF155 [25]. We suspect that the difference in colocalization is due to the specific role the ATPase module plays in SWI/SNF complex identity and genomic targeting. In mammals, there are three types of SWI/SNF subcomplexes, each that contain distinct SWI/SNF subunits that define their identity and location of action across the genome [19]. In cancer cells with dual ATPase loss, however, the retained SWI/SNF complexes contain most, but not all, defining SWI/SNF subunits, and without the ATPase present, the subcomplexes lack their unique functional characteristics, including genomic localization [15]. We suspect that because BRG1 remains present in MRT, BRG1 may facilitate targeting of SWI/SNF to MYC-bound genes, which would be lost in SCCOHT cells. Additional studies in more BRG1-null cell lines, particularly those that lack BRG1 but retain the second ATPase, BRM, will be very informative for telling if SCCOHT cells are the exception or the rule when it comes to MYC-SWI/SNF chromatin interactions.

When we employed the dTAG system [34] to deplete MYC in BIN-67 cells, we found that there is a prominent decrease in DNA repair gene expression (Figure 3 and Figure 4). DNA repair pathways are critical for preserving genome integrity and are routinely dysregulated in cancer [48]. The overexpression of MYC is known to induce DNA damage and replicative stress as part of its suite of oncogenic functions, which promotes genomic instability that drives tumorigenesis [52]. However, MYC also binds to promoters of double-strand DNA break genes and mismatch repair genes, thereby facilitating expression of the genes needed to protect the cell from any overly destructive damage, which works to assist cell survival [44,53]. SCCOHT and MRT tumors are notorious for their high genome stability and low mutational burden, including any genetic lesions that would increase *MYC* expression [23,54]. Considering this, it is interesting to speculate based on our data that MYC contributes to the enhanced genomic stability inherent to SCCOHT and rhabdoid tumors by acting as a regulator of DNA repair gene expression.

The insights gleaned from this study imply that loss of a single SWI/SNF subunit sets in motion a cascade of diverse molecular changes that together drive all aspects of cancer formation, including DNA repair alterations. Consistent with this, the reintroduction of BRG1 oppositely regulates DNA repair gene expression by reducing the levels of these gene products. Intriguingly, this effect occurs in a catalytic activity-independent manner (Figure 5a–d) and therefore is not related to the ability of SWI/SNF to actively remodel chromatin. It is also not overtly related to the binding of ATPase-deficient BRG1 at DNA repair genes (Figure 5e) but instead may be due to the impairment of *MYC* expression (Figure 5f). The mechanisms by which ATPase-deficient BRG1 regulates *MYC* expression in unknown, but because ATPase-deficient BRG1 is detected within the *MYC* promoter (Figure 5g) and the reintroduction of ATPase-deficient BRG1 can reestablish the genomic localization of SWI/SNF complexes [15], we favor the idea that interactions between restored SWI/SNF and transcriptional regulators at the *MYC* gene modulate *MYC* expression without the requirement of altered DNA accessibility. It is also possible that the reintroduction of BRG1 can directly regulate MYC functionality rather than solely working through an effect on *MYC* expression levels, similar to what has been found with SNF5 and MYC in MRT. Future experiments performed at shorter timepoints following the reintroduction of BRG1 will be necessary to separate out direct and indirect effects of SWI/SNF on MYC activities.

Our identification of an MYC-driven DNA repair gene signature in SCCOHT cell lines led us to interrogate our findings in tumor samples. Strikingly, we found that genes involved in multiple DNA repair pathways are upregulated in SCCOHT cancers (Figure 6). These data are consistent with recent studies using a gene expression analysis and a single cell RNA-seq analysis of SCCOHT tumors [55,56]. Although MYC does not directly bind to as many DNA repair genes as it regulates (Figure 4d), one key DNA repair gene we identify as an MYC target—and is substantially upregulated in SCCOHT tumors—is *ATR* (Figure 4d and Figure 6). The ATR kinase is a master regulator of DNA repair pathway function as it lies at the top of an extensive signaling network that allows the cell to respond to replicative stress and DNA damage [57]. Cancer cells tend to depend on ATR for the maintenance of genome integrity and the loss of ATR can downregulate the expression of other DNA repair proteins [57]. As such, MYC could control multiple DNA repair pathways by fine-tuning the expression of *ATR*, which then would feed into the various pathways that protect DNA-centric processes. Importantly, higher *ATR* expression correlates with worse prognosis in *SMARCA4*-null tumors, and the genetic and chemical inhibition of ATR without any other perturbation impairs SCCOHT colony formation [56]. These effects were inhibited in the presence of BRG1, pointing to a dependency on ATR that exists only in the absence of this subunit.

From a clinical perspective, the increased expression of DNA repair genes in SCCOHT and MRT tumors (Figure 6 and Figure 7) may have implications for the treatment of these diseases. In cancer, MYC-driven increases in DNA repair gene expression are typically associated with resistance to DNA damaging agents. Therefore, our findings could explain why chemotherapy treatments for SCCOHT patients, which are centered on the inclusion of platinum-based DNA damaging agents, rarely lead to positive outcomes [3,58]. There is also a possibility that the gene signature we identify here highlights potential vulnerabilities that can be exploited for therapy. Inhibitors of poly-ADP-ribose polymerase (PARP), which work to prevent base excision repair and non-homologous end joining, are a new treatment for ovarian cancers [59]. In SCCOHT, patient-derived xenograft models show impaired growth in response to the PARP inhibitor, Talazoparib. This effect is amplified by combining Talazoparib with other targeted therapies [56], suggesting that a combination of DNA repair inhibitors with additional anticancer agents results in increased therapeutic benefit. In support of this, in malignant rhabdoid tumor xenografts, Talazoparib modestly impairs tumor growth, but when combined with a DNA alkylating agent substantially improved antitumor activity is achieved [60]. Moreover, the PARP inhibitor Rucaparib is moderately effective at decreasing colony formation and growth of atypical teratoid rhabdoid tumors in xenograft mouse models, but treatment with this agent sensitizes cells to radiation and the combination of Rucaparib and radiation extends mouse survival [61]. At this time, we do not know if upregulation of DNA repair gene expression prevents therapies that target DNA repair from functioning at their full potential as single agents. But, these recent reports do reveal that dampening DNA repair activity does enable synergistic effects with other therapies. With this in mind, we suggest that PARP inhibitors—or other DNA repair protein inhibitors such as those that target ATR—may help achieve a beneficial outcome for these deadly diseases when used in combination with chemotherapies, radiotherapy, or other targeted anticancer agents.

## 5. Conclusions

Overall, our study set out to interrogate the relationship between BRG1 and MYC in SCCOHT. We discovered that while MYC binds to a fraction of active promoters in BIN-67 cells, the number of genes MYC regulates—whether directly or indirectly—are numerous and conspicuously converge on a DNA repair gene expression program. The majority of these genes are oppositely regulated BRG1, which is due at least in part to the non-catalytic activity mechanism that BRG1 exerts on *MYC* expression. We identify a DNA repair gene signature that is upregulated in both SCCOHT and rhabdoid tumors, which we posit influences how these tumors respond to treatment in the clinic. This study has created a framework in which to investigate the MYC-BRG1 connection in additional BRG1-null cancers and discovered a unique commonality among two different SWI/SNF-altered cancers that can be connected back to MYC.

## Data Availability

The sequencing data generated in this study are deposited on GEO under GSE298397. The BIN-67 ChIP-seq data for BAF155 and K785R BRG1 before and after the reintroduction of BRG1 were previously published under GSE117734 [15]. The BIN-67 BRG1/K785R BRG1 RNA-sequencing results used for comparison to those generated in this study were reported as a supplemental file in [15]. The RNA-sequencing data for the 10 SCCOHT tumors were collected and analyzed in [14] and are under dbGaP: phs001528.v1.p1, GSE109919 and GSE216801. Additional data are available upon request.

## Supplementary Materials

The following supporting information can be downloaded at https://www.mdpi.com/article/10.3390/cancers17132255/s1, Figure S1: Uncropped Western blots; Figure S2: Additional analysis of MYC-regulated genes in engineered BIN-67 cells; Figure S3: Expression data for genes involved in oxidative phosphorylation; Table S1: Significant gene expression changes (FDR < 0.05) identified in V1-treated cells compared to NEG-treated cells; Table S2: Output of gene set enrichment analysis using genes identified in V1 vs. NEG analysis against MSigDB Hallmark datasets; Table S3: Comparison of DNA repair and oxidative phosphorylation gene expression from SCCOHT tumors as compared to HGSC tumors. Reference [62] is cited in the supplementary materials.

## Abbreviations

The following abbreviations are used in this manuscript:

SCCOHT	Small cell carcinoma of the ovary, hypercalcemic type
HGSC	High-grade serous carcinoma
DMSO	Dimethyl sulfoxide
NEG	dTAG^V^-1-NEG (negative chemical control)
V1`	dTAG^V^-1 (degrader chemical)
CUT&RUN	Cleavage under targets and release using nuclease
ChIP	Chromatin immunoprecipitation
TSS	Transcription start site
H3K4me3	Histone 3 lysine 4 trimethylation
ATR	Ataxia telangiectasia and Rad3-related protein
BRCA1	Breast cancer type 1 susceptibility protein
PARP	Poly-ADP-ribose polymerase
BRG1	Brahma-related gene 1
SNF5	Sucrose Non-Fermenting 5
BRM	Brahma
MRT	Malignant rhabdoid tumor
