# Supplementary material for: MYC Regulates a DNA Repair Gene Expression Program in Small Cell Carcinoma of the Ovary, Hypercalcemic Type
Source: Cancers (Basel). 2025 Jul 7;17(13):2255. doi: 10.3390/cancers17132255 (PMC12248706; doi:10.3390/cancers17132255)
Supplement: Supplementary file 1 [file cancers-17-02255-s001.zip › Supplementary Material.pdf]

Supplementary Materials

MYC regulates a DNA repair gene expression program in small cell carcinoma of the ovary, hypercalcemic type

Evans JR et al.

Included figures and tables:

Figure S1. Uncropped Western blots. Uncropped blots used to generate the primary figures, along with an associated colorimetric image showing protein markers.

Figure S2. Additional analysis of MYC regulated genes in engineered BIN-67 cells. (**a**) mRNA from engineered BIN-67 cells treated with DMSO, 500 nM dTAG^{V}-1-NEG (NEG) or 500 nM dTAG^{V}-1 (V1) for 24 hours was analyzed by examining the levels of the indicated genes. GAPDH mRNA levels were quantified and used for normalization (n = 4 biological replicates, error bars are standard error of the mean). (**b**) Gene ontology analysis was performed using genes that increase in expression following MYC depletion as determined in the V1 vs. NEG analysis (FDR < 0.05, fold change > 1.5). Analysis was performed using ShinyGO 0.82 [1] and “GO Biological Process”. (**c**) KEGG pathway analysis was performed using genes that decrease in expression following MYC depletion as determined in the V1 vs. NEG analysis (FDR < 0.05, fold change < -1.5). Analysis was performed using ShinyGO 0.82 and “KEGG pathway database” [2]. (**d**) Network plot showing connectedness of gene categories from (c). Groups that share ≥ 20% of genes are connected by the nodes. Darker green indicates higher significance of enrichment and larger nodes correlate with size of the gene set.

Figure S3. Expression data for genes involved in oxidative phosphorylation. Over 20 oxidative phosphorylation (OXPHOS) genes that are regulated by MYC in BIN-67 cells were separated and categorized based on their known roles as core or accessory proteins involved in OXPHOS [3]. RNA expression levels are shown for normal ovary tissue, small cell carcinoma of the ovary hypercalcemic type (SCCOHT) tumors, and age-matched high-grade serous ovarian carcinoma (HGSC) cancers. Boxes mark the 25th to 75th percentiles with a middle line indicating the median value and whiskers extending from highest to lowest data points. Student’s unpaired *t*-test was performed on data from SCCOHT tumors and normal ovary tissue, and *p*-values are shown. Black *p*-values indicate a significant increase in SCCOHT tumors versus normal ovary tissue and red *p*-values indicate a significant increase in normal ovary tissue versus SCCOHT tumors. Statistical analysis was also performed on data from SCCOHT tumors versus HGSC and those *p*-values are in Table S3.

Excel files:

Table S1. Significant gene expression changes (FDR < 0.05) identified in V1-treated cells compared to NEG-treated cells. Sheet one lists genes that significantly decreased in expression upon MYC depletion and sheet two includes genes that significantly increased in expression upon MYC depletion.

Table S2. Output of gene set enrichment analysis using genes identified in V1 vs. NEG analysis against MSigDB Hallmark datasets.

Table S3. Comparison of DNA repair and oxidative phosphorylation gene expression from SCCOHT tumors as compared to HGSC tumors. DNA repair genes are in blue text and oxidative phosphorylation genes are in red. A positive value for the "Difference" column indicates expression is higher in SCCOHT than HGSC and a negative difference value indicates expression of that gene is higher in HGSC versus SCCOHT. *p*-values were obtained by employing an unpaired student *t*-test for each gene in Prism GraphPad software v10.0.2.

References

1. Ge, S.X.; Jung, D.; Yao, R. ShinyGO: a graphical gene-set enrichment tool for animals and plants. *Bioinformatics* **2020**, *36*, 2628-2629, doi:10.1093/bioinformatics/btz931.
2. Kanehisa, M.; Furumichi, M.; Sato, Y.; Ishiguro-Watanabe, M.; Tanabe, M. KEGG: integrating viruses and cellular organisms. *Nucleic Acids Res* **2021**, *49*, D545-D551, doi:10.1093/nar/gkaa970.
3. Frederick, M.; Skinner, H.D.; Kazi, S.A.; Sikora, A.G.; Sandulache, V.C. High expression of oxidative phosphorylation genes predicts improved survival in squamous cell carcinomas of the head and neck and lung. *Sci Rep* **2020**, *10*, 6380, doi:10.1038/s41598-020-63448-z.

Uncropped blot Figure 1a, GAPDH

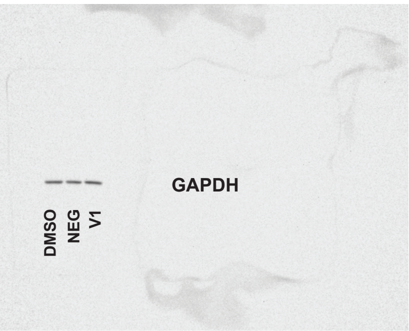

Uncropped blot Figure 1a, MYC

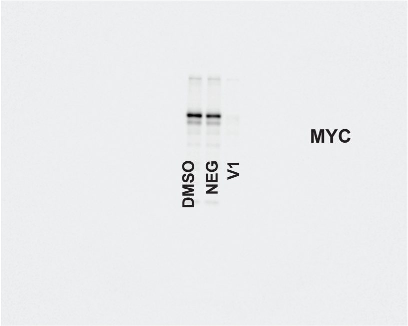

Uncropped blot Figure 1a, SNF5 and BAF155

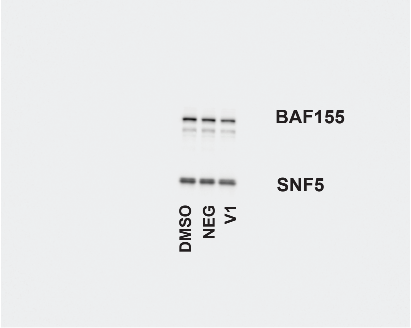

Uncropped blot Figure 1a
with Ladder
merge for GAPDH antibody

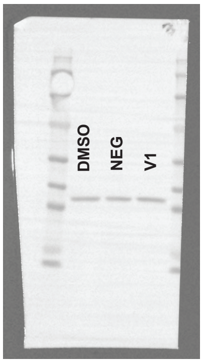

Uncropped blot Figure 1a
with Ladder
merge for MYC antibody

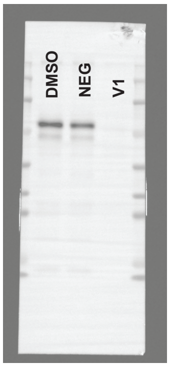

Uncropped blot Figure 1a
with Ladder
merge for SNF5 and BAF155 antibody

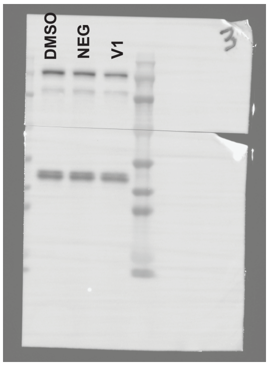

Uncropped blot Figure 1b, GAPDH

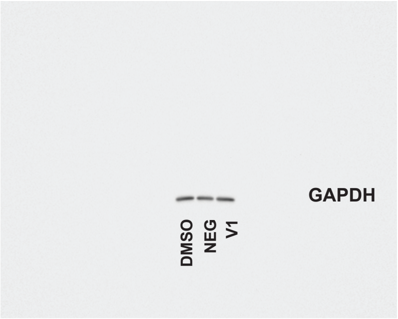

Uncropped blot Figure 1b, MYC

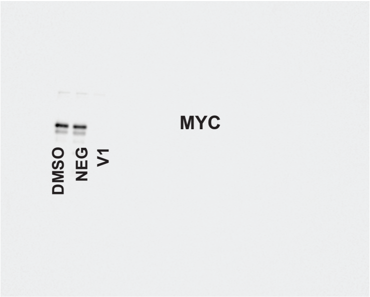

Uncropped blot Figure 1b, SNF5 and BAF155

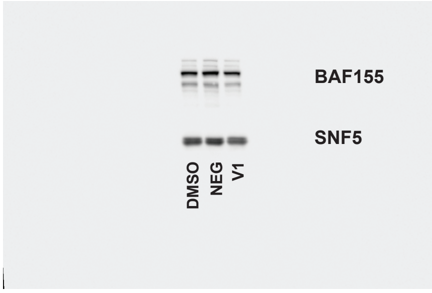

Uncropped blot Figure 1b
with Ladder
merge for GAPDH antibody

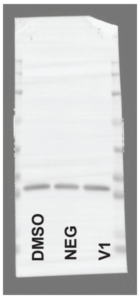

Uncropped blot Figure 1b
with Ladder
merge for MYC antibody

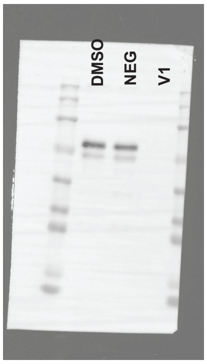

Uncropped blot Figure 1b
with Ladder
merge for SNF5 and BAF155 antibody

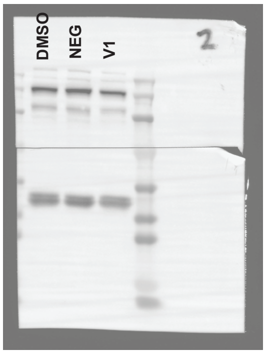

Uncropped blot Figure 4f, GAPDH

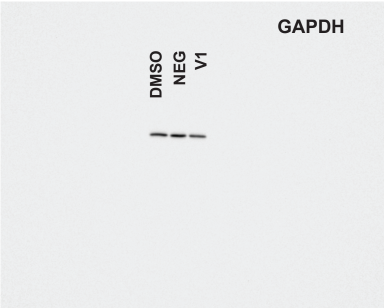

Uncropped blot Figure 4f
with Ladder
merge for GAPDH antibody

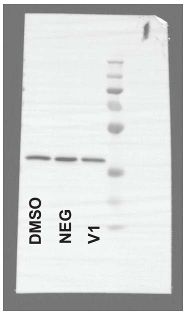

Uncropped blot Figure 4f, MYC

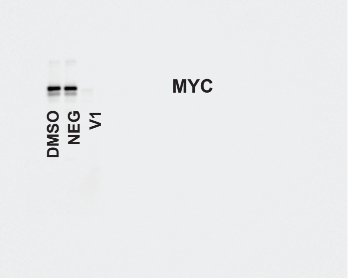

Uncropped blot Figure 4f, ATR

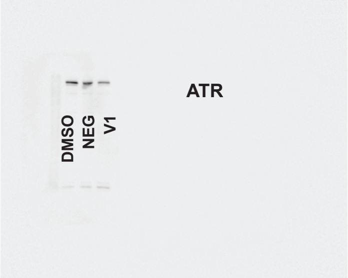

Uncropped blot Figure 4f, RAD51

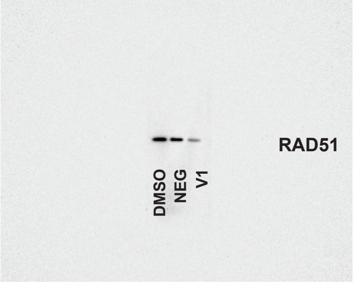

Uncropped blot Figure 4f
with Ladder
merge for MYC antibody

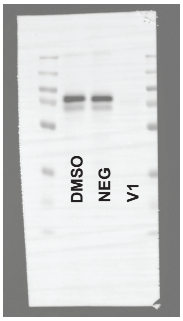

Uncropped blot Figure 4f
with HiMarkLadder
merge for ATR antibody

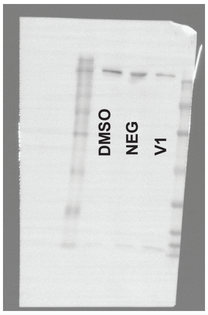

Uncropped blot Figure 4f
with Ladder
merge for RAD51 antibody

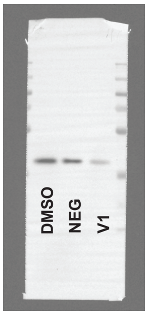

Uncropped blot Figure 4g, GAPDH

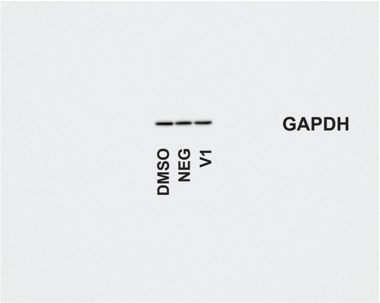

Uncropped blot Figure 4g, MYC

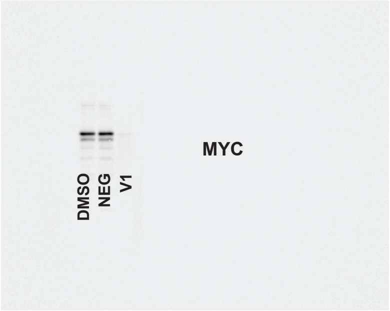

Uncropped blot Figure 4g, ATR

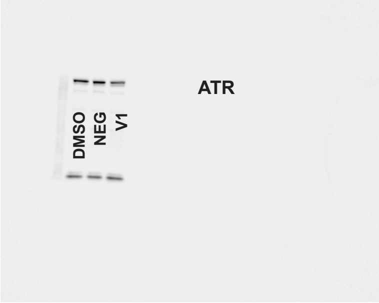

Uncropped blot Figure 4g, RAD51

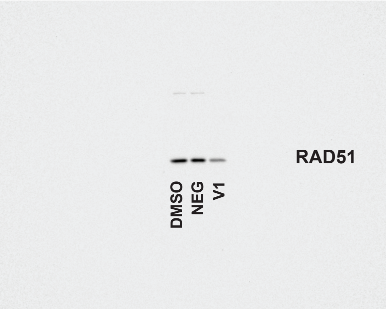

Uncropped blot Figure 4g
with Ladder
merge for GAPDH antibody

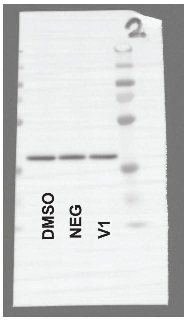

Uncropped blot Figure 4g
with Ladder
merge for MYC antibody

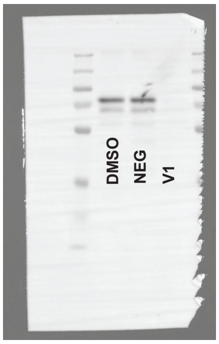

Uncropped blot Figure 4g
with HiMarkLadder
merge for ATR antibody

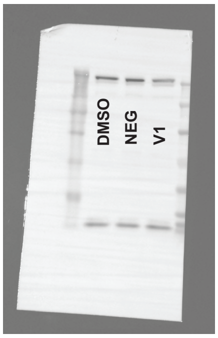

Uncropped blot Figure 4g
with Ladder
merge for RAD51 antibody

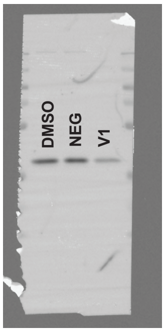

Uncropped blot Figure 4h, GAPDH, MYC, and RAD51

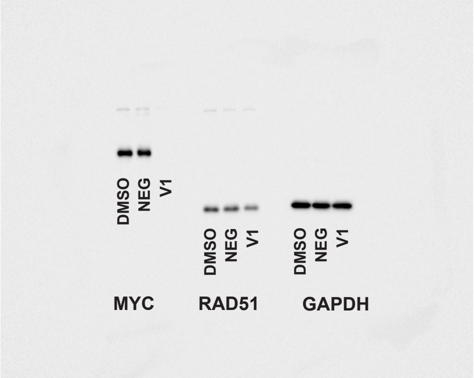

Uncropped blot Figure 4h, ATR

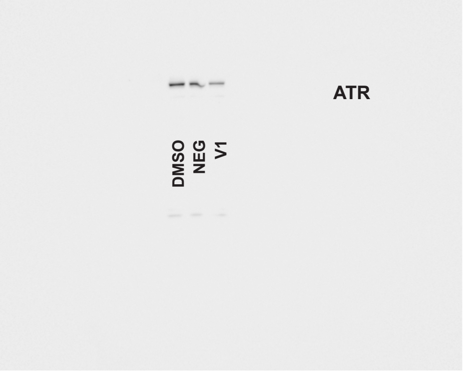

Uncropped blot Figure 4h
with HiMarkLadder
merge for ATR antibody

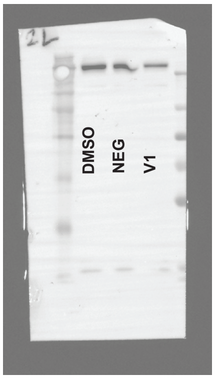

Uncropped blot Figure 4h
with Ladder
merge for MYC, GAPDH, and RAD51 antibody

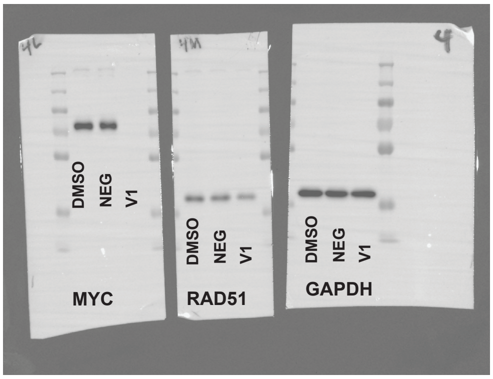

Figure S1. Uncropped Western blots. Uncropped blots used to generate the primary figures, along with an associated colorimetric image showing protein markers.

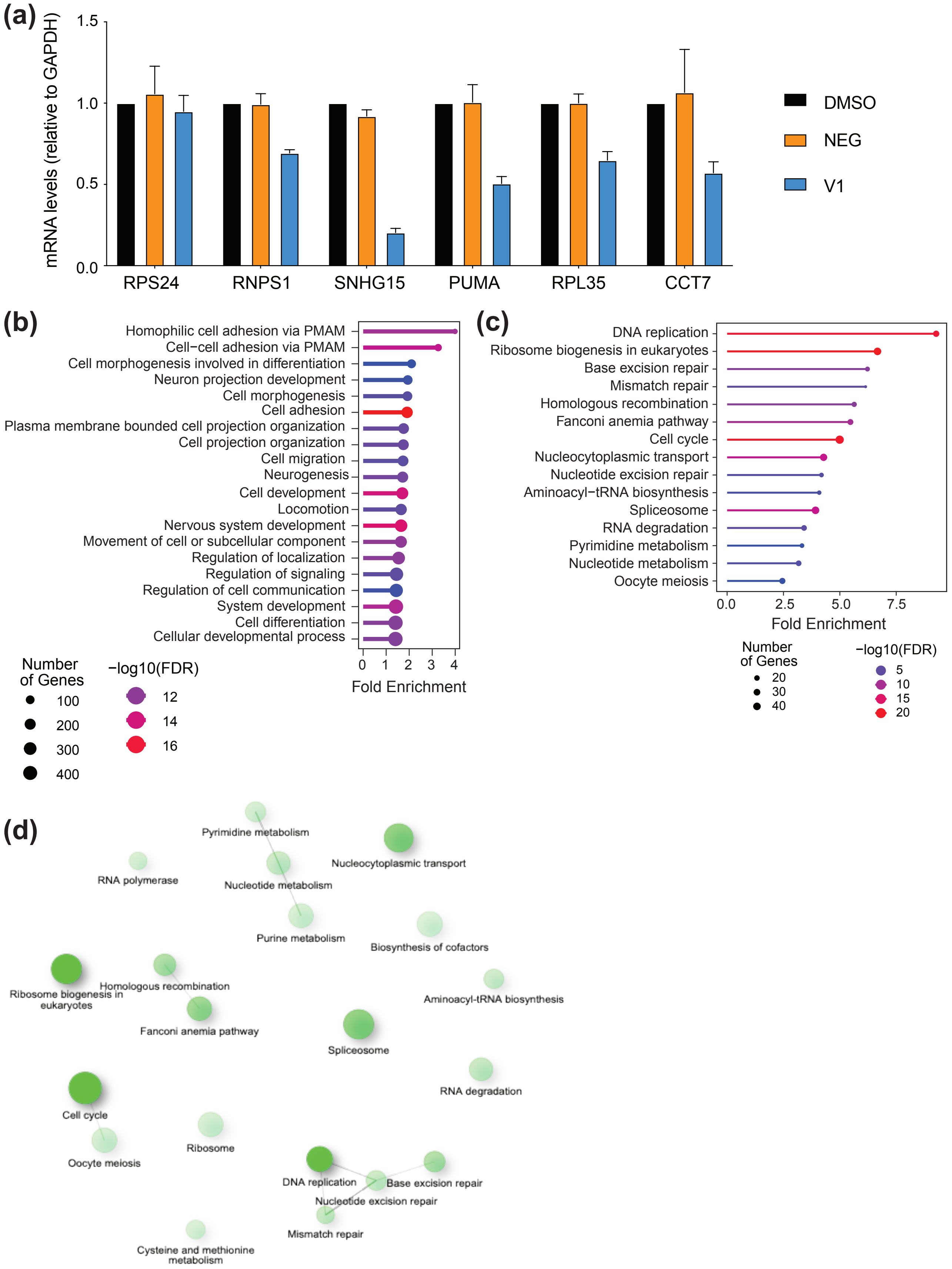

(a)
mRNA levels (relative to GAPDH)
1.5
1.0
0.5
0.0
RPS24
RNPS1
SNHG15
PUMA
RPL35
CCT7
DMSO
NEG
V1
(b)
Homophilic cell adhesion via PMAM
Cell−cell adhesion via PMAM
Cell morphogenesis involved in differentiation
Neuron projection development
Cell morphogenesis
Cell adhesion
Plasma membrane bounded cell projection organization
Cell projection organization
Cell migration
Neurogenesis
Cell development
Locomotion
Nervous system development
Movement of cell or subcellular component
Regulation of localization
Regulation of signaling
Regulation of cell communication
System development
Cell differentiation
Cellular developmental process
0 1 2 3 4
Fold Enrichment
Number of Genes
100
200
300
400
−log10(FDR)
12
14
16
(c)
DNA replication
Ribosome biogenesis in eukaryotes
Base excision repair
Mismatch repair
Homologous recombination
Fanconi anemia pathway
Cell cycle
Nucleocytoplasmic transport
Nucleotide excision repair
Aminoacyl−tRNA biosynthesis
Spliceosome
RNA degradation
Pyrimidine metabolism
Nucleotide metabolism
Oocyte meiosis
0.0 2.5 5.0 7.5
Fold Enrichment
Number of Genes
20
30
40
−log10(FDR)
5
10
15
20
(d)
Pyrimidine metabolism
Nucleocytoplasmic transport
RNA polymerase
Nucleotide metabolism
Purine metabolism
Biosynthesis of cofactors
Ribosome biogenesis in eukaryotes
Homologous recombination
Aminoacyl-tRNA biosynthesis
Fanconi anemia pathway
Spliceosome
RNA degradation
Cell cycle
Oocyte meiosis
Ribosome
DNA replication
Base excision repair
Nucleotide excision repair
Mismatch repair
Cysteine and methionine metabolism

Figure S2. Additional analysis of MYC regulated genes in engineered BIN-67 cells. (a) mRNA from engineered BIN-67 cells treated with DMSO, 500 nM dTAGV-1-NEG (NEG) or 500 nM dTAGV-1 (V1) for 24 hours was analyzed by examining the levels of the indicated genes. GAPDH mRNA levels were quantified and used for normalization (n = 4 biological replicates, error bars are standard error of the mean). (b) Gene ontology analysis was performed using genes that increase in expression following MYC depletion as determined in the V1 vs. NEG analysis (FDR < 0.05, fold change > 1.5). Analysis was performed using ShinyGO 0.82 [1] and "GO Biological Process". (c) KEGG pathway analysis was performed using genes that decrease in expression following MYC depletion as determined in the V1 vs. NEG analysis (FDR < 0.05, fold change < -1.5). Analysis was performed using ShinyGO 0.82 and "KEGG pathway database" [2]. (d) Network plot showing connectedness of gene categories from (c). Groups that share ≥20% of genes are connected by the nodes. Darker green indicates higher significance of enrichment and larger nodes correlate with size of the gene set.

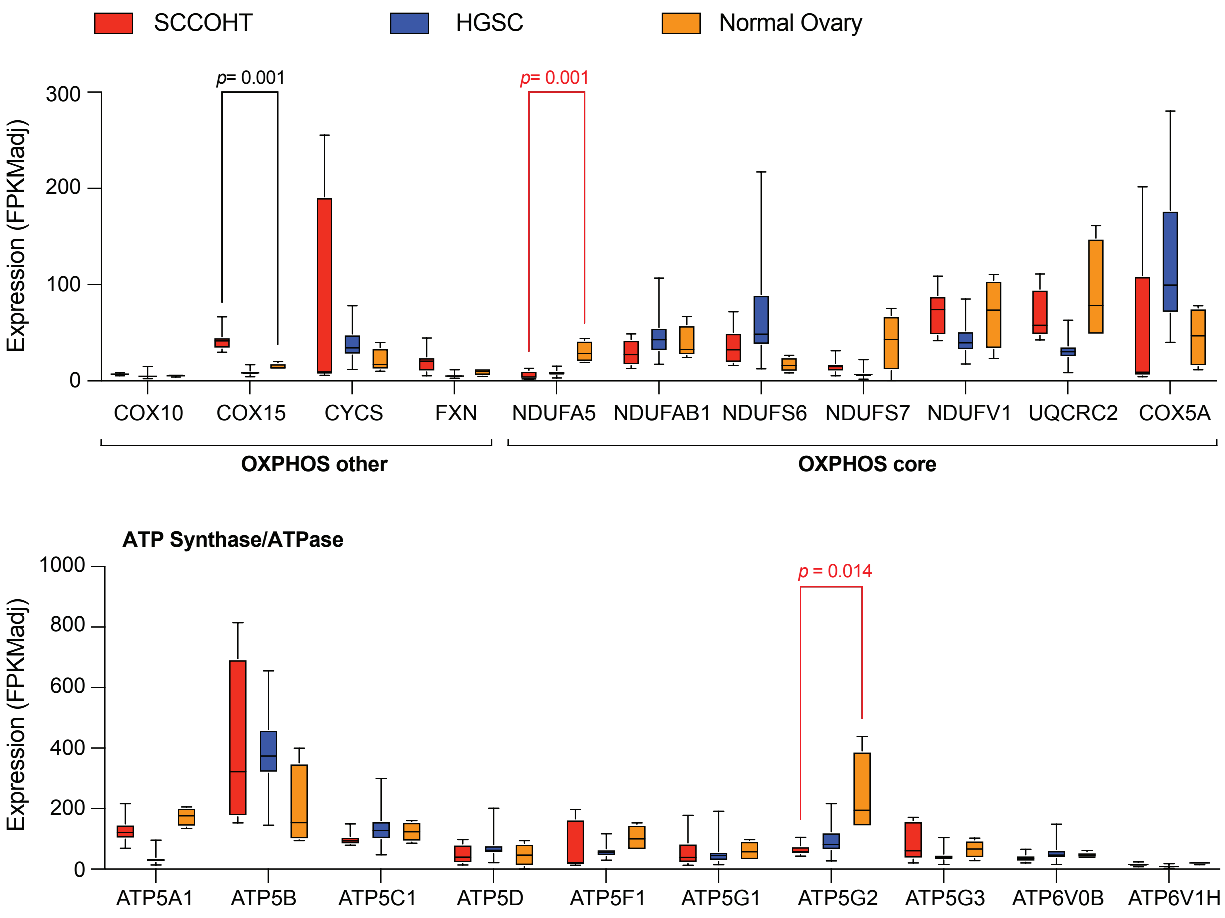

Figure S3. Expression data for genes involved in oxidative phosphorylation. Over 20 oxidative phosphorylation (OXPHOS) genes that are regulated by MYC in BIN-67 cells were separated and categorized based on their known roles as core or accessory proteins involved in OXPHOS [3]. RNA expression levels are shown for normal ovary tissue, small cell carcinoma of the ovary hypercalcemic type (SCCOHT) tumors, and age-matched high-grade serous ovarian carcinoma (HGSC) cancers. Boxes mark the 25th to 75th percentiles with a middle line indicating the median value and whiskers extending from highest to lowest data points. Student's unpaired *t*-test was performed on data from SCCOHT tumors and normal ovary tissue, and *p*-values are shown. Black p-values indicate a significant increase in SCCOHT tumors versus normal ovary tissue and red *p*-values indicate a significant increase in normal ovary tissue versus SCCOHT tumors. Statistical analysis was also performed on data from SCCOHT tumors versus HGSC and those *p*-values are in Table S3.
